# Real-time methylation-specific PCR for the evaluation of methylation status of MGMT gene in glioblastoma

**DOI:** 10.18632/oncotarget.25543

**Published:** 2018-06-12

**Authors:** Masaki Yoshioka, Tomoo Matsutani, Ayaka Hara, Seiichiro Hirono, Takaki Hiwasa, Masaki Takiguchi, Yasuo Iwadate

**Affiliations:** ^1^ Department of Neurological Surgery, Chiba University Graduate School of Medicine, Chiba, Japan; ^2^ Department of Biochemistry and Genetics, Chiba University Graduate School of Medicine, Chiba, Japan

**Keywords:** glioma, MGMT, methylation, real-time PCR, MSP

## Abstract

The methylation status of the O6-methylguanine-DNA methyltransferase (MGMT) gene is a strong predictor for the efficacy of temozolomide chemotherapy and survival periods. However, the correlation between the extent of methylation and the difference in survival times has not been fully clarified. Simple and quantitative evaluations of the methylation status in the promotor region of the MGMT gene are expected to be worldwide standardized diagnostics. We applied real-time semi-quantitative methylation-specific polymerase chain reaction (SQ-MSP) of the MGMT gene promoter region to 84 glioblastoma patients. The SQ-MSP result showed that the ΔCt value, which represents the difference between uCt and mCt (uCt value – mCt value), is inversely correlated with overall survival. With adequate cutoff setting, this assay showed that those patients suffering from a tumor with low ΔCt (methylated) survived significantly longer than those having tumors with high ΔCt (un-methylated). The most significant difference was observed when the cutoff was set at a ΔCt of 2. Using this cutoff point, the result of MGMT immunohistochemical analysis was also significantly correlated with the methylation status examined with real-time SQ-MSP. These results collectively show that MGMT promoter methylation status actually affects patients’ survival and protein expression depending on its methylation level, and the extent of methylated CpGs would be better assessed with real-time SQ-MSP than with the standard gel-based MSP. This method is cost- and labor-saving compared with pyrosequencing, and significantly contributes to the accurate and objective prediction of patient survival.

## INTRODUCTION

Glioblastoma is one of the most malignant cancers of the central nervous system. Standard treatment includes radiotherapy and temozolomide chemotherapy after surgery. Temozolomide is an alkylating agent which causes DNA damage by delivering a methyl group to purine bases of DNA (O6-guanine; N7-guanine and N3-adenine) and induces apoptosis [1]. However, O6-methylguanine-DNA methyltransferase (MGMT), which is a DNA repair protein, reverses alkylation at the O6 position of guanine, thus decreasing the cytotoxic effects of alkylating drugs such as temozolomide. Silencing the MGMT gene by promoter methylation results in decreased MGMT expression and improves the effects of temozolomide [2–3].

Recent studies have shown that MGMT gene promoter methylation status, either methylated or un-methylated, is related to patient survival; patients with high levels of MGMT gene promoter methylation status are expected to survive longer [4–5]. It is important to accurately evaluate the methylation status of the MGMT gene promoter in clinical decision making for treatment selection and in the development of novel therapies including MGMT silencing by tumor-targeted siRNA delivery [6–8]. There are several methods that can be used to analyze MGMT promoter methylation, including pyrosequencing (PSQ), methylation-specific polymerase chain reaction (MSP), methylation-specific multiplex ligation-dependent probe amplification (MS-MLPA), and Infinium Methylation BeadChip technology [9–14]. Many researchers consider PSQ to be the best method because it can be used to analyze the methylated ratio of each target CpG site separately [10–12]. However, it is difficult to interpret PSQ data into clinically-relevant information correlating with MGMT protein expression or patient survival. Furthermore, this method is cost- and labor-intensive, with a necessity for initial investment in the novel sequencer, which prevents this assay from being a world-wide standard essential for the clinical routine and clinical trials. An easier and more quantitative method for analyzing the MGMT promoter methylation status is needed in order to obtain clinically-relevant information in daily medical practice.

MSP is a cost- and labor-saving method compared with PSQ and can be performed with simple equipment. However, the prevalent MSP is subjective in evaluation because of a lack in quantitative data formation. To facilitate objective evaluation of MSP data, real-time MSP using SYBR-Green technology offers an easy way to semi-quantitatively express the methylation status without requiring the laborious gel electrophoresis [15–17]. In this study, we showed that the real-time semi-quantitative MSP (SQ-MSP) is an effective method for evaluating the methylation status of the MGMT gene promoter for the accurate prediction of patient survival.

## RESULTS

### Real-time MSP

The most appropriate way to read data is to look at the dissociation curve first and check whether there is a methylation primer specific peak at 81° C. When there is such a at 81° C in the dissociation curve, there are methylated tumor cells in the sample. If not, there are no methylated tumor cells in the sample, and those having no peak at 81° C in dissociation curve were considered to be unmethylated tumors. In the amplification curve, we can see how much DNA is being amplified. The number of PCR cycles at which the amplification curve exceeds a certain threshold―fluorescence was 0.1 dRn in this study― is defined as the Ct value. The threshold should be determined at the value where the slope of the amplification curve is not an exponential amplification but a stable amplification. Thus, mCt means that the amplification curve of methylated MGMT primer at the PCR cycle and uCt corresponds to unmethylated MGMT.

**Figure 1:**
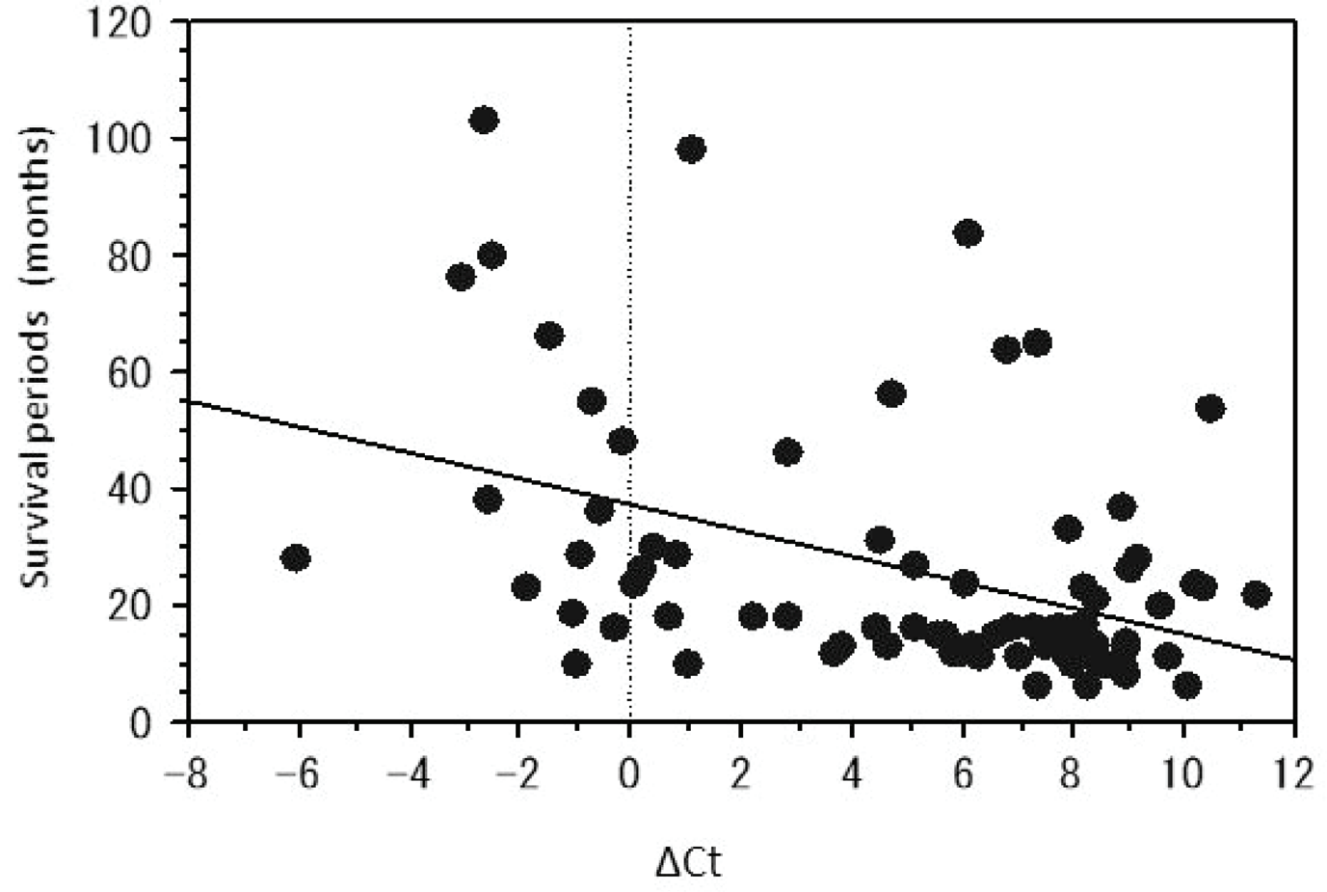
The scatter diagrams of the relationship between the ΔCt values and the survival periods of the patients with glioblastoma having the peak at 81° C The survival period is inversely proportional to the ΔCt obtained by the real-time semi-quantitative methylation specific PCR (SQ-PCR).

In order to quantify the methylation status, we evaluated the ΔCt value, which represents the difference between uCt and mCt (uCt value – mCt value). The ΔCt values of the tumors having no peak at 81° C in dissociation curve were between 4 and 10. In contrast, the tumors with a large peak at 81° C have a relatively small ΔCt, mainly around 0. These tumors were considered to be in the methylated group. A scatter diagram was obtained, showing the relationship between the ΔCt value and the survival period in months (Figure 1). The scatter diagram shows that the survival time of the patients with unmethylated tumors tends to be short, whereas the samples with a small ΔCt value contain many long-term survivors. There was a negative correlation between the ΔCt value and patient survival time (R = 0.361, *p* = 0.0010).

### ΔCt cutoff for the most precise survival prediction

**Figure 2:**
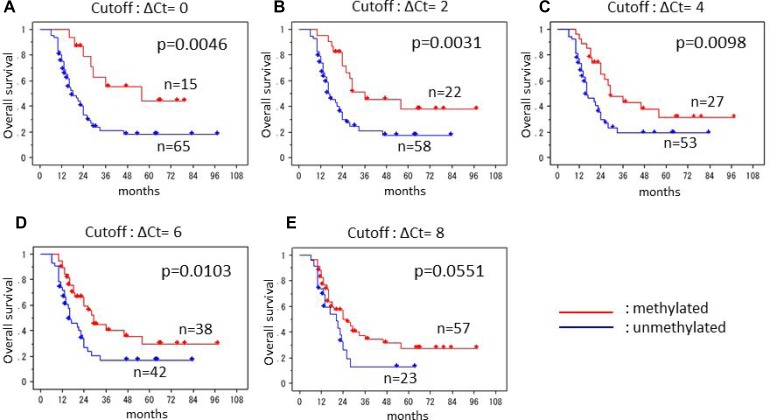
Kaplan-Meier survival curves of the patients with glioblastoma Five cutoffs of ΔCt values were applied to separate methylated and unmethylated groups; (**A**) cutoff ΔCt of 0, (**B**) cutoff ΔCt of 2, (**C**) cutoff ΔCt of 4, (**D**) cutoff ΔCt of 6, (**E**) cutoff ΔCt of 8. The smallest *p*-value was obtained at the cutoff off 2.

In order to clinically apply this research, we would like to classify those samples having a peak at 81° C into either the methylated group or the unmethylated group by ΔCt value. The smaller the ΔCt value is, the greater the proportion of methylated cells and the greater the extent of the methylated region in each cell. Therefore, we set five cutoffs to distinguish between the methylated and unmethylated groups, and compared the survival period between the two groups in each cutoff (Figure 2). When the ΔCt cutoff value was set at 0, 2, 4, 6, and 8, all of the comparisons except with a cutoff of 8 yielded statistically significant differences. The smallest *p*-value was obtained at the cutoff off 2.

### Comparison of ΔCt and gel electrophoresis of MSP products

**Figure 3:**
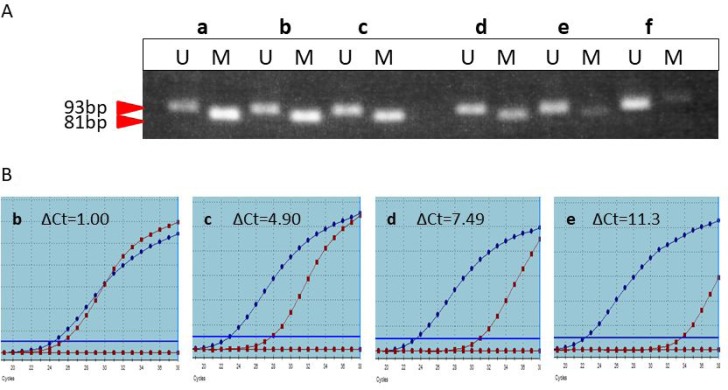
Correlation of the band intensity on the gel (**A**) and the dissociation curves at each ΔCt (**B**). Only when the ΔCt was approximately over 10 or in the sample having no peak at 81° C, no apparent band was visualized in the gels.

To extend real-time MSP to the worldwide -routine clinical practice, it is important to visualize the band intensity at each ΔCt (Figure 3). Although the ΔCt values varied from 1 to 8, there were distinct methylated bands on the gels. When the ΔCt was approximately over 10 or in the sample having no peak at 81° C, no apparent band was visualized in the gels. This indicates that a wide range in the proportion of methylated cells or in the extent of the methylated regions in each cell results in a band formation in the methylated lane. Actually, the functional threshold of MGMT promoter methylation may be within in the range of ΔCt yielding the methylated band on the gel.

### Correlation of real-time MSP and MGMT protein expression

We ultimately examined the correlation of the ΔCt from real-time MSP results and the MGMT protein expression evaluated by immunohistochemistry. Although the valid evaluation of immunohistochemical analyses is not easy, judgement of negative results usually does not give significant discordance. We regarded a negative immunohistochemical result as a surrogate target, and examined the correlation with real-time MSP results; unmethylated results linked with protein-positive was judged as positive-predictive values (PPV) and methylated results linked with protein-negative was judged as negative-predictive values (NPV). When the methylation status was determined only by the presence of a methylation primer specific peak at 81° C in the dissociation curve, the PPV was 93% and the NPV was 58%. There was a highly significant correlation in the methylation status of the MGMT promoter region and MGMT protein expression (*p* < 0.00001, Fisher exact test). However, when the judgement of the methylation status included a ΔCt with a cutoff value of 2, the PPV was 92% and the NPV was 70%. The PPV was almost the same, but the NPV was remarkably improved. When ΔCt with cutoff values of 4 and 6 were used, the PPVs were 94 and 91%, and the NPVs were 53 and 47%, respectively (Table 1). Overall, a ΔCt with a cutoff value of 2 yielded the most excellent predictive values for MGMT protein expression.

**Table 1:** PPV and NPV according to the cutoff value in ΔCt

ΔCt value	PPV (%)	NPV (%)	*p*-value
None	93	59	<0.0001
2	92	71	<0.0001
4	94	54	<0.0001
6	100	53	<0.0001

Abbreviations: PPV: Positive predictive value

NPV: Negative predictive value

## DISCUSSION

In the present study, we clearly showed that the information obtained from real-time SQ-MSP concerning MGMT promoter methylation status is highly correlated with the survival period of the GBM patients treated with the standard Stupp’s protocol. The results of real-time SQ-MSP are also highly correlated with the protein expression examined by immunohistochemistry. The heterogeneous methylation patterns of respective cases were adequately evaluated by SQ-MSP, which led to its high correlation with both patient survival and MGMT protein expression.

Although some contradictory results have been reported [18, 19], the correlation of MGMT promoter methylation status with patient survival is well demonstrated. In previous studies, it has been shown by many authors that the outcome is better in patients with MGMT promoter methylation, which is usually determined by gel-based MSP in the clinical routine [2, 3, 20–24]. However, the relationship of MGMT methylation status to MGMT protein expression is controversial, with many researchers reporting that there is no significant correlation [18, 22, 23, 25]. Methylation status is usually so heterogeneous within a tumor, not in an all-or-none manner, that the way to quantitatively or semi-quantitatively assess methylation status has long been discussed [4, 5, 9–13]. The present result shows that there is a positive relationship between MGMT promoter methylation and protein expression when the methylation status is adequately examined with SQ-MSP. It is a cardinal rule of biology that promoter methylation beyond a certain amount has a definite effect on silencing gene expression.

Human MGMT promoter regions include 98 CpG sites and only a portion of the sites are used for standard MSP or pyrosequencing [26]. The extent of MGMT promoter methylation is different among tumor cells [27–29], and normal cells usually contaminate all samples, all of which affect the real-time MSP results. The minimal methylation level at the CpG sites that is required to suppress MGMT protein expression has not been fully elucidated. The criterion obtained by utilizing cell lines is not easily adapted into clinics because of the complexity of human GBM samples intermingled with non-neoplastic cells such as microglia and macrophages. That is because the quantitative assessment of MGMT methylation status with an appropriate threshold setting focusing on patient outcomes is quite important for reliable clinical application. The real-time SQ-MSP result is actually highly correlated with the MGMT immunohistochemical results as well as the patient outcomes, which can be attributed to the adequate threshold setting. Although the primers used in this study are designed to encompass the well-correlated regions with MGMT protein expression, it is not clear if these CpG sites best reflect the status of expression. We should consider the possibility that another combination of methylation sites would affect the MGMT activity of the tumors.

In this study, the methylation status was evaluated with the real-time SQ-MSP, introducing the ΔCt value to “relatively” quantify the MGMT promoter methylation. The result is that patient survival time is negatively correlated with ΔCt. The difference in survival periods between two groups divided by ΔCt was most significant when it was set at 2 (*p* = 0.0031). This cutoff value also led to the most significant correlation of the methylation status with the protein expression. Since gel electrophoresis of the MSP products showed both methylated and unmethylated bands of varying degrees, quantification of the extent of methylation is important for the precise clinical application of the MSP results.

Indeed, when the ΔCt value was 2, the rate of MGMT promoter methylation was 0.275, which is considerably lower than that in previous reports of around 0.4 [3, 27–29]. The gel-based MSP included tumors with slightly-methylated MGMT promoter that were not sufficiently methylated to repress MGMT protein expression [30–32]. That is partly because MSP is prone to false-positive results and there were some negative reports for the predictive and prognostic values of MGMT methylation status [22]. Although regulation of MGMT protein expression is affected by many other factors, it was shown here that the promoter methylation is clearly involved in the inactivation of the MGMT gene, resulting in a clinically-significant shortage of the protein. Overall, the evaluation of MGMT promoter methylation using real-time SQ-MSP is quite useful for predicting the efficacy of temozolomide in clinical practice. However, although the ΔCt precisely represents the methylation level of the MGMT promoter, it should be noted that the value is not an absolute numerical value that quantifies the methylated CpG islands.

The real-time SQ-MSP examined here is cost-efficient and can easily be introduced into routine work without a large initial financial investment, making the evaluation of MGMT promoter methylation status more reliable than the gel-based MSP which is currently assumed to be the gold standard for MGMT methylation analysis.

## MATERIALS AND METHODS

### Patients and tissue specimens

Eighty-four patients with GBM were included in this study. The background characteristics are summarized in Table 2. IDH1 immunohistochemistry was positive in only three cases. All tissue samples were obtained from patients at the Chiba University Hospital under a protocol approved by the Ethics Committee of the Chiba University Graduate School of Medicine, and informed consent was obtained from the patients or their guardians. The histopathological diagnoses for all specimens were confirmed by two independent neuropathologists, according to the criteria established by the World Health Organization. The patients with GBM were homogeneously treated with 60 Gy local-field irradiation and concurrent temozolomide. The initial chemotherapy protocol was administered precisely according to Stupp’s regimen. All tumor specimens investigated were obtained at the time of the first surgery for each patient. A portion of each sample was fixed in 10% formaldehyde and embedded in paraffin, and the remainder of the sample was immediately frozen in liquid nitrogen and stored at −80° C until protein extraction.

**Table 2:** Patients characteristics

Characteristics	*N* (%)
Sex	
Male	43 (51%)
Female	41 (49%)
Age	
<60	36 (43%)
60**≤**	48 (57%)
Karnofsky Performance Status	
70**≤**	37 (44%)
<70	47 (56%)
Surgery	
Total resection	36 (43%)
Non-total resection	48 (57%)
MGMT immunohistochemistry	
Negative	20 (27%)
Positive	53 (73%)
IDH1 gene status	
Wild-type	72 (94%)
Mutant	5 (6%)

### DNA extraction and bisulfite conversion

In the first step of the experiment, we extracted DNA from the frozen samples of glioblastoma stored in liquid nitrogen. We used NucleoSpin Tissue for DNA isolation. After the DNA was isolated, double—stranded DNA concentrations were measured for bisulfite conversion. We mixed the isolated DNA with pure water, and adjusted it to 5 µg/ml. Bisulfite conversion was performed with the EZ DNA Methylation-Gold kit. During the bisulfite treatment, unmethylated cytosine is converted to uracil while methylated cytosine remains unchanged.

### Real time methylation-specific polymerase chain reaction (Real time MSP)

Brilliant II SYBR Green qPCR Master Mix and two types of primers were used for MSP. Primers were designed for the methylation-favorite site of the MGMT promoter sequences. One type of primer is specific to fully methylated sequences, and recognizes unconverted cytosine during bisulfite treatment, whereas the other type of primer is for fully unmethylated sequences, binding to uracil which is changed from cytosine. The primer sequences are as follows [33]: mMGMT forward 5′-TTTCGACGTTCGTAGGTTTTCGC-3′, mMGMT reverse 5′-GCACTCTTCCGAAAACGAAACG-3′, uMGMT forward 5′-TTTGTGTTTTGATGTTTGTAGGTTTTTGT-3′, and uMGMT reverse 5′-AACTCCACACTCTTCCAAAAACAAAACA-3′. Real-time PCR was performed at 95° C for 15 min, then 38 cycles of 95° C for 50 s, 59° C for 50 s and 72° C for 50 s, followed by a final step at 72° C for 10 min. The PCR mixture contains bisulfite-converted DNA, 4 × SYBR Green PCR Master Mix, and each forward and reverse primer. All of the procedures were done in duplicate to confirm repeatability and they were averaged following analysis. A feature of real-time MSP is that we can get two important pieces of data: a dissociation curve and an amplification curve. The dissociation curve shows the temperature at which the DNA undergo a change of state from double strands to a single strand, so one can see if the targeting site of the MGMT promoter gene is amplified. On the other hand, from the amplification curve, we can observe how much DNA is being amplified over time.

### Gel electrophoresis of MSP products

The real-time MSP products were evaluated using standard 3% agarose gel-electrophoresis stained with ethidium bromide and they were visualized under ultraviolet illumination to be compared with the real-time MSP results. The bands were present on the gel at 93 bp and 81 bp for unmethylated and methylated MGMT promoter sequence, respectively.

### Immunohistochemistry for MGMT protein and IDH1 mutation

Formalin-fixed paraffin-embedded tissue sections were deparaffinized inxylene on microscopic slides. Antigen retrieval was performed by microwaving the sections in 10 mM citric acid buffer (pH 7.2). The primary antibodies used in this study were: anti-human IDH1-R132H monoclonal antibody (1:100, IBL Co., Ltd, Gumma, Japan) and anti-MGMT monoclonal antibody MT3.1 (1:200, Chemicon, Inc., Temecula, CA). The samples were incubated with the primary antibody overnight, followed by incubation with a biotinylated secondary antibody (1:500, Dako, Tokyo, Japan). The bound antibodies were visualized using the avidin biotin peroxidase complex method and diaminobenzidine tetrachloride (Santa Cruz Biotechnology, Inc.). To evaluate IDH1 staining, strong cytoplasmic staining in any number of cells was scored as positive. For MGMT scoring, the positive cells in a 200 × field (minimum of 1,000 nuclei) were counted, and the labeling index was expressed as a percentage of the labeled tumor cells. MGMT protein expression ≥10% was considered positive.

### Statistical analysis

Progression-free survival (PFS) was calculated from the date of initial surgery until the first sign of radiological progression, death, or last follow-up. Overall survival (OS) was also calculated from the date of initial surgery until the date of death or last follow-up. Using StatView software (SAS Institute Inc., Cary, NC), the Kaplan-Meier method was used to estimate the survival rates, and the Cox-Mantel log-rank test was applied to compare the survival differences among the patients. The other potential prognostic variables were age, extent of surgery, Karnofsky performance status score, and MGMT protein expression. Multivariate analysis was performed with commercially available software using the Cox proportional hazards regression model (SPSS, Inc., Chicago, IL).
